# Concomitant dislocation of the tarsometatarsal and metatarsophalangeal joints of the second toe (floating second metatarsal): a case report

**DOI:** 10.1186/1757-1626-2-39

**Published:** 2009-01-10

**Authors:** Mahmood Karimi Mobarake, Alireza Saied, Elisabeth Baron

**Affiliations:** 1 Kerman University of Medical Sciences, Kerman, Iran; 2 Orthopedics Dept, Dr. Bahonar Hospital, Kerman Neuroscience research center, Kerman, Iran; 3 Brigham & Women's Hospital, 75 Francis Street, Boston, MA 02116, USA

## Abstract

When examining patients with injuries of the tarsometatarsal joint, the physician must pay attention to the foot as a whole.

An extremely rare foot injury has been described in which axial and compressive forces cause simultaneous dislocation of the tarsometatarsal joint and the metatarsophalangeal joint of the same or adjacent ray. The following is a report of one of these rare injuries.

We will also discuss probable mechanism and diagnosis of this rare traumatic injury.

## Case presentation

**Figure 1:**
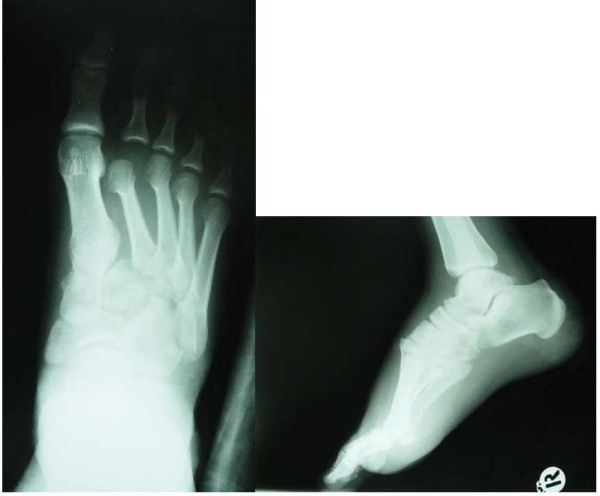
**Anteroposterior, oblique and lateral views of the right foot of the patient on arrival**. (The date, seen on the lateral view is the time of slide preparation.)

A 22 year old man presented to our emergency department after involvement in a head-on motor vehicle collision at an unknown speed. The patient was unrestrained in the right front passenger seat at the time of the accident. History (including the past medical history) and physical exam revealed no significant findings except for severe pain, tenderness, and swelling in the right foot and distal leg. Radiographs revealed fracture of the right tibia, Lisfranc fracture and dislocation (fracture of the cuboid and lateral dislocation of the four lateral tarsometatarsal joints), and dislocation of the second metatarsophalangeal joint (Figure 1).

**Figure 2:**
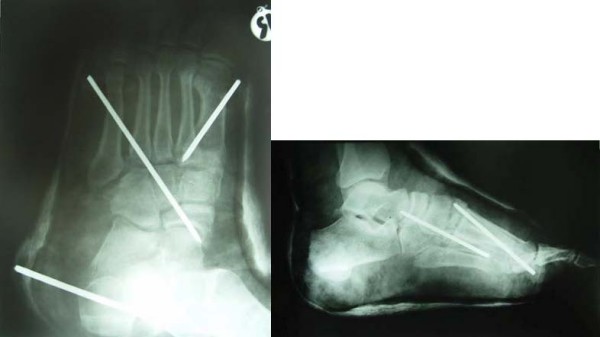
**Postoperative radiograms, successful reduction of the MTP and incomplete reduction of the TMT joints**.

After counseling the patient on both open and closed treatment options, the patient opted for closed methods and opposed open reduction entirely (he was not comfortable with open surgery at all). Under general anesthesia, closed reduction of the metatarsophalangeal joint was performed. Closed reduction of the tarsometatarsal joints and percutaneous fixation were then performed. The tibia fracture was treated by placing pins in the calcaneus and proximal tibia and splinted with a plaster cast. Post-operative radiographs confirmed the successful reduction of the metatarsophalangeal joint, but the reduction of the Lisfranc joint, although near anatomic was incomplete (Figure 2). The patient was unwilling to consent to surgery and was discharged the following day after receiving intravenous antibiotics and an uneventful hospital course. He was subsequently lost to follow up and the pins and cast removed at another institution.

**Figure 3:**
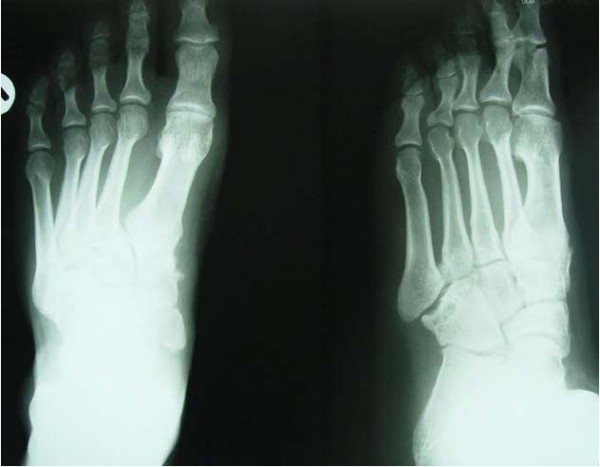
**One and half years after the initial trauma, early evidence of DJD has appeared in the Lisfranc joints, especially the cuboid-fifth metatarsal joint**.

The patient was contacted one and a half years after the initial trauma and agreed to return for follow up. At that time he was essentially asymptomatic; he was not limping and had full range of motion of the foot and ankle. Radiographs of the patient are shown in figure 3.

## Discussion

Lisfranc joint injuries should be considered in every patient involved in a motor vehicle accident and retrospective studies have shown that these injuries are missed in up to 20% of cases on initial evaluation [1]. One of the mechanisms of Lisfranc joint injuries is axial loading associated with compression forces [2]. Dislocations of the lesser metatarsophalangeal joints are produced by axial forces usually during hyperextension of the toes [2]. This type of trauma is not common and reduction of the dislocation of these joints is easily achieved with traction, dorsiflexion, and plantarflexion, but it has been reported that closed reduction is successful in 50% of cases [2]. Simultaneous dislocation of the tarsometatarsal and metatarsophalangeal joints in a single ray is extremely rare, and to the best of our knowledge, only seven cases have been previously reported [3-9].

English first reported an association between Lisfranc joint and metatarsophalangeal joint dislocations [10]. He described a patient who had concomitant dislocations of the tarsometatarsal joint of a ray and the metatarsophalangeal joint of the adjacent ray. English used the term "linked toe metatars" to describe the condition and proposed the theory that traction on the soft tissue, especially the first dorsal interosseous muscle after dislocation of the tarsometatarsal joint, causes the metatarsophalangeal joint to dislocate. He based his theory on the observation the reduction of the metatarsophalangeal joint was impossible before reduction of the tarsometatarsal joint.

However, the opposite has also been proposed [11]. An alternative theory is that most metatarsophalangeal dislocations are caused by axial loading during dorsiflexion of the toes and if axial loading continues with plantarflexion of the foot, the tarsometatarsal joint will dislocate as well.

Either of these theories could have been the mechanism of injury to our patient, as the third tarsometatarsal joint was dislocated as well, but it was noted that the metatarsophalangeal dislocation was easily reducible ***before ***the tarsometatarsal joint which disagrees with English's theory.

Our patient was essentially asymptomatic one and a half years following his injury. Considering his young age, inadequate reduction of the Lisfranc joint, and early radiographic evidence of degenerative joint disease, complications including early arthropathic pain and limping are likely to develop [2,11].

Associated injuries occur in 32–68% of Lisfranc joint fractures and dislocations [11,12] and essentially any bone of the foot or ankle may be involved [13]. The diagnosis of metatarsophalangeal joint dislocation can be made easily with good quality radiograms, but it can be quite easily missed with either overpenetration of the film or poor quality films [14]. The anteroposterior view may show only subtle widening of the joint and the lateral view can be misleading due to overlapping of the metatarsal heads [15]. All of these emphasize the fact that the examining physician must pay adequate attention when examining patients with either of these injuries.

## Competing interests

The authors declare that they have no competing interests.

## Consent

Written informed consent was obtained from the patient for publication of this case report and accompanying images. A copy of the written consent is available for review by the Editor-in-Chief of this journal.

## Authors' contributions

M.K.M wrote the discussion and introduction parts of the article and performed most of the review of literature. A.R.S. wrote the case presentation and performed some of review of the literature. He managed the case and recognized it as suitable for publication. E.B. edited the final manuscript and prepared it as acceptable for journal.
